# Incidence of natal and neonatal teeth: a 15-year retrospective study from the Greater Poland voivodeship

**DOI:** 10.1186/s12903-025-07173-x

**Published:** 2025-11-24

**Authors:** Aleksandra Szymczak, Michał Suchodolski, Dominika Forszt, Aneta Olszewska, Magdalena Roszak, Elżbieta Paszyńska

**Affiliations:** 1 Department of Integrated Dentistry, Poznan University of Medical Sciences, Poznań, Poland; 2 Greater Poland Center of Digital Medicine, Poznan University of Medical Sciences, Poznań, Poland; 3 Department of Risk Group Dentistry, Poznan University of Medical Sciences, Poznań, Poland; 4 Department of Orthodontics and Temporomandibular Disorders, Poznan University of Medical Sciences, Poznań, Poland; 5 Department of Pathophysiology, Poznan University of Medical Sciences, Poznań, Poland

**Keywords:** Natal teeth, Newborn, Prevalence, Retrospective studies, Tooth, Deciduous

## Abstract

**Background:**

Natal and neonatal teeth are deciduous teeth present at birth or erupting within the first 30 days of life, respectively. They are a rare dental phenomenon with unclear etiology. Their presence can lead to clinical complications such as feeding difficulties or risk of aspiration.

This study aimed to assess the incidence of natal and neonatal teeth in the Polish population over a 15-year period, analyze temporal trends in their occurrence and evaluate potential associations with perinatal variables, including newborn sex, gestational age, and maternal age.

**Methods:**

A retrospective analysis was conducted using medical records coded as K00.6 (Dentia praecox) submitted to the Greater Poland National Health Fund between 2007 and 2022. Birth data were obtained from the Central Statistical Office. Statistical analysis included binary logistic regression to assess trends over time and associations with delivery timing and maternal age. Multinomial logistic regression was applied to evaluate changes in the number of pathological teeth per case. The Fisher–Freeman–Halton test with Monte Carlo simulation was used to examine sex-based differences. A literature review of studies published between 2010 and 2024 was performed for global comparison.

**Results:**

Out of all live births in the region, 471 cases were identified (0.08% prevalence), with natal teeth comprising 93.8% of cases. The incidence increased from 0.039% in 2007 to 0.134% in 2022.

Logistic regression revealed statistically significant increases in on-time deliveries and post-term births among affected cases. Maternal age under 20 and between 20 and 35 years was significantly associated with a higher probability of children having natal or neonatal teeth. A notable shift from single to multiple-teeth cases over time was observed. No significant differences in prevalence by sex were found. Comparative analysis indicated variation in global prevalence, likely due to methodological and demographic differences.

**Conclusions:**

The prevalence of natal and neonatal teeth in the Polish population has increased over the past 15 years, with significant associations observed with maternal age and birth timing. These findings underscore the importance of continued surveillance and standardized data reporting. Future international studies with consistent methodologies and statistical analysis are needed to clarify etiological factors and guide clinical management of affected infants.

## Background

Teeth that emerge within 30 days are referred to as neonatal teeth while those present in the oral cavity from birth are termed natal teeth [1]. The occurrence of this condition varies between 1:1000 to 1:30000 [2]. Moreover, natal teeth occur three times more frequently in comparison with neonatal ones [3] and are predominantly found in the anterior region of the mandible [4]. In fact, 85% of natal/neonatal teeth appear in the anterior section of the mandible, followed by the anterior section in the maxilla and the canine area in the mandible. The least common occurrence of this condition is observed in the canine area in the maxilla as well as the upper and lower molar region [5]. Generally, natal, and neonatal teeth represent a manifestation of prematurely erupted primary incisors, and in approximately 1 to 10% of cases, these natal/neonatal teeth are supernumerary [6]. Numerous factors have been attributed to this phenomenon, yet the etiology has not been precisely understood [5].

Investigations have elucidated several potential origins, which include superficial location of tooth buds, systemic conditions, environmental toxins, hereditary predisposition, and genetic syndromes [3]. Other factors encompass poor maternal condition, febrile incidents during pregnancy, disruptions in the endocrine system particularly related to thyroid, gonads and pituitary, congenital syphilis [7]. Subsequent proposed explanation involves osteoblastic activity around the tooth buds [3]. A positive family history is correlated with 15% of natal/neonatal teeth cases [8]. According to the literature, natal or neonatal teeth could be a manifestation of congenital syndromes, such as Hallermann–Streiff, Ellis–van Creveld, Jadassohn–Lewandowsky, Rubinstein-Taybi, Pfeiffer syndromes, craniofacial dysostosis steatocystoma multiplex and adrenogenital syndromes [9].

The emergence of natal/neonatal teeth can affect both the child and the mother. Common ramifications associated with this condition are ulcerations of sublingual region as well as frenulum, and injuries of mother’s breasts while feeding a baby [4]. Those teeth, irritating the mother’s nipple and the baby’s oral mucosa, contribute to inconvenience during suckling and thus feeding difficulties [10]. Damage derived from natal and neonatal teeth to an infant’s oral mucosa is called Riga-Fede disease [11]. The consequence of long-term presence of Riga-Fede disease leads to tongue injury, malnutrition, and growth reduction [12]. The development of the injury embraces recurring trauma caused by contact with anterior teeth during repeated tongue movements [13]. Moreover, lack of proper root formation of the natal/neonatal teeth leads to them being affixed to the oral mucosa [10]. Teeth hypermobility poses the risk of swallowing or aspiration by a child [14].

Aim of this study was to present the incidence of natal and neonatal teeth in recent years among the Polish population, investigate temporal trends in their occurrence and to analyze potential statistical associations between the presence of these teeth and selected perinatal factors, including the sex of the newborn, gestational age at birth, and maternal age.

To further explore the risk factors for natal and neonatal teeth and compare their incidence globally, a literature review was also conducted.

## Methods

### Study design and data sources

The presented study was a retrospective analysis based on medical records submitted by dental and medical service providers, focusing on cases diagnosed with ICD-10 code K00.6 (Dentia praecox), as reported to the Greater Poland National Health Fund (NFZ). The data covers the occurrence of natal and neonatal teeth in the Wielkopolskie Voivodeship between 2007 and 2022 (Table 1).

**Table 1:**
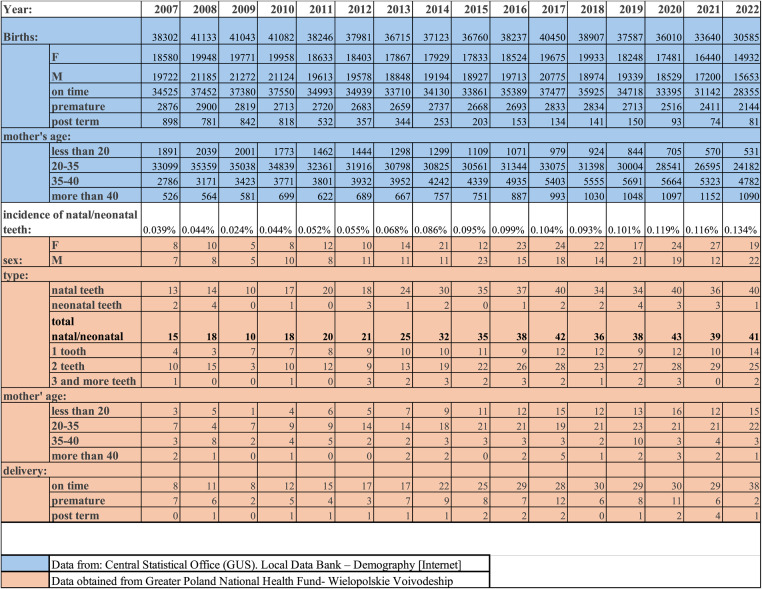
Data on natal and neonatal teeth provided by Greater Poland National Health Fund (NFZ) and birth information obtained from Central Statistical Office (GUS)

### Study population and variables

Available data were categorized based on the patient's sex, mother's age, time of delivery, as well as the type of preterm dentition- natal or neonatal and were presented separately for each monitored year. Corresponding demographic information representing the entire population of the voivodeship was sourced from the Central Statistical Office (GUS) website [15], providing the basis for calculating incidence rates and conducting further statistical analyses.

### Statistical analysis

Statistical analysis of birth-related pathology trends from 2007 to 2022 was performed using Python (version 3.13.3) and R (version 4.3.2). Binary logistic regression was applied to model the annual trends in the odds of specific outcomes, such as delivery timing and dental pathology, based on maternal age groups. Multinomial logistic regression in R assessed changes in the severity of dental pathology, comparing single-tooth vs. multi-tooth cases over time. The Fisher–Freeman–Halton test with Monte Carlo simulation was used to explore gender differences in pathology distribution.

### Literature review

A comparative literature search was conducted by reviewing original research papers published in PubMed and Medline databases. The search included only English language retrospective studies published from 2010 to 2024 and following keywords were used for the search: “natal teeth”, “neonatal teeth”. Two authors (S.A., F.D.) independently screened the abstracts, selecting studies that provided information on the incidence of natal and neonatal teeth in the study population. Eventually, the data obtained from included papers was evaluated in table 2.

**Table 2:**
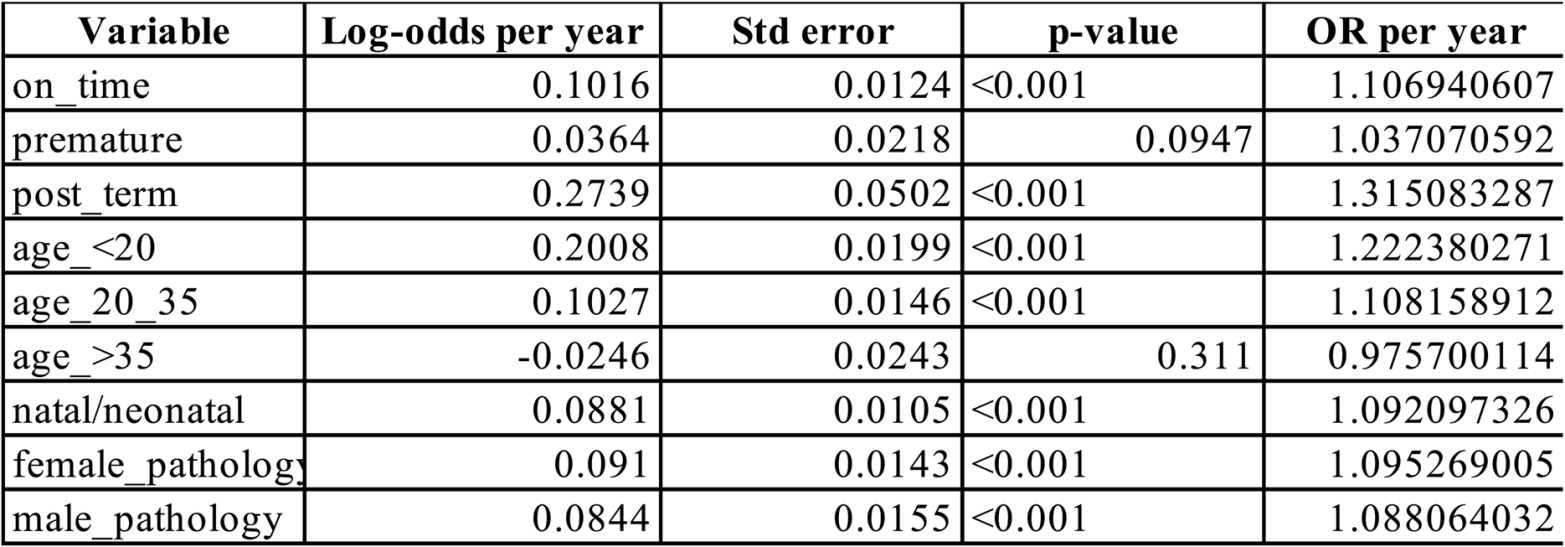
Binary logistic regression results

*Binary logistic regression over year using binomial log-likelihood optimization using Python 3. 13. 3

## Results

### Prevalence of natal and neonatal teeth

Using data obtained from the Central Statistical Office on the number of births within the voivodeship, along with information from the National Health Fund regarding the incidence of natal and neonatal teeth, the prevalence of this phenomenon between 2007 and 2022 was determined to be 0.08%. The total number of cases was 471 with natal teeth being far more prevalent compared to neonatal teeth (442 vs. 29 cases).

### Incidence trends (2007–2022)

**Fig. 1:**
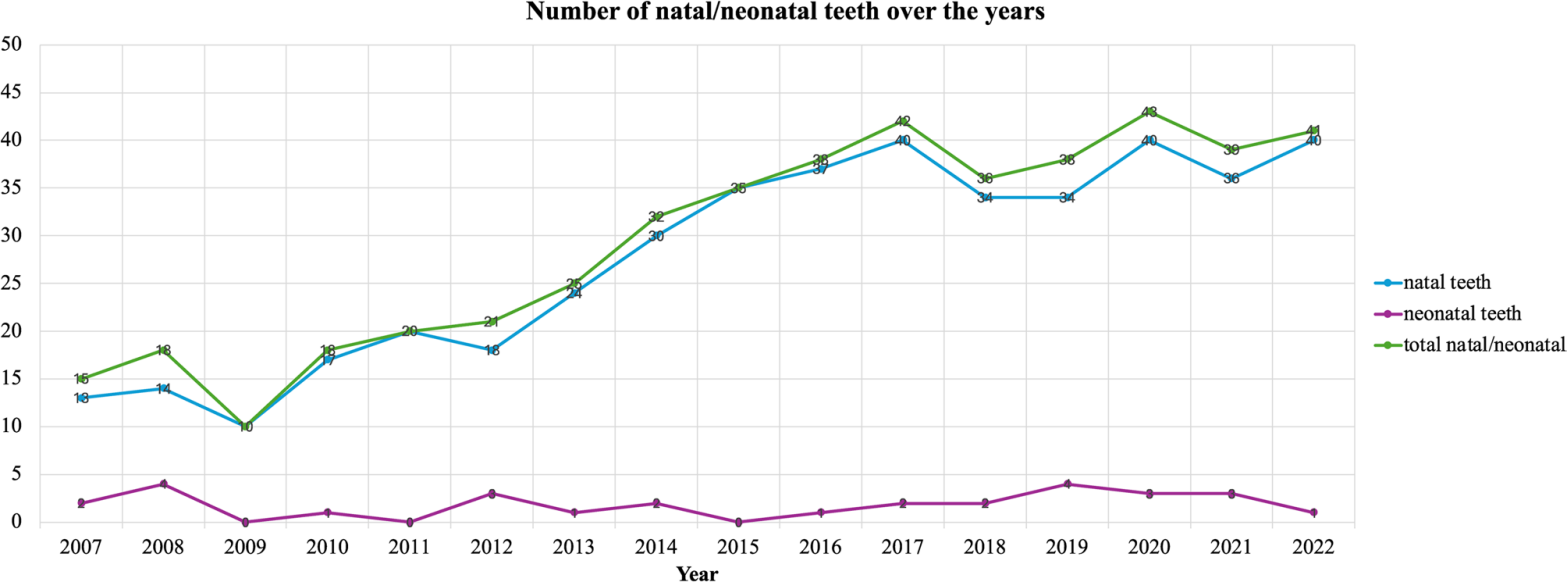
The annual number of natal and neonatal teeth cases from 2007 to 2022

Over the 15-year period from 2007 to 2022 the incidence of both natal and neonatal teeth demonstrated a consistent upward trend with minor fluctuations. The lowest recorded incidence was 0.024%, while the highest incidence reached 0.134%. In the initial years, the percentage remained relatively low, starting at 0.039% and increasing slightly to 0.055%. Increases were observed in the later years and the incidence eventually rose to 0.134% in 2022 (Fig. 1).

All factors associated with the presence of natal and neonatal teeth, along with the annual incidence rates, are presented in Table 1.

### Statistical analysis

#### Birth-related pathology trends

Statistical analysis of birth related pathology trends from 2007 to 2022 was conducted using both Python (ver. 3.13.3) and R (ver. 4.3.2). Binary logistic regression was applied to model annual trends in the odds of specific outcomes including timing of delivery of children with dental pathology (on-time, premature, post-term), maternal age groups of children with pathologies and presence of natal/neonatal teeth. Odds ratios and p-values were derived from binomial log-likelihood functions using the Python programming environment. This has revealed statistically significant increases over time in the odds of on-time delivery (OR ≈ 1.107, *p* < 0.001), post-term births (OR ≈ 1.315, *p* < 0.001) and dental pathology in both males (OR ≈ 1.088, *p* < 0.001) and females (OR ≈ 1.095, *p* < 0.001) (Table 2).

#### Maternal age influence

Maternal age was categorized into four groups: less than 20 years, 20–35 years, 35–40 years, and more than 40 years and the analysis showed that maternal age significantly influenced the likelihood of children having natal and neonatal teeth. Specifically, mothers aged under 20 (OR ≈ 1.222, *p* < 0.001) and those aged 20–35 (OR ≈ 1.108, *p* < 0.001) had a significantly higher chance of having children with natal/neonatal teeth. However, for mothers aged over 35, no significant trend was observed (OR ≈ 0.976, *p* = 0.31), meaning that maternal age over 35 did not appear to impact the occurrence of natal/neonatal teeth in their children (Table 2).

#### Changes in severity of pathology and gender differences in its distribution

Moreover, to assess changes in severity, multinomial logistic regression was performed in R using “nnet” library. Comparing the relative odds of one vs. multiple pathological teeth over time has revealed a statistically significant decline in single-tooth cases and an increase in multi-tooth pathology (OR ≈ 1.029/year, *p* < 0.001), suggesting a valid shift in counts (Table 3). Additionally, the Fisher–Freeman–Halton test with Monte Carlo simulation (100,000 replicates) using R environment found no significant difference in the temporal distribution of natal/neonatal teeth between males and females (*p* = 0.368).

**Table 3:**
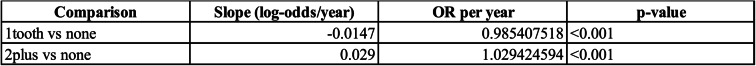
Multinomial logistic regression results

*Multinomial logistic regression (R version 4. 3. 2 with 'nnet::multinom' function) with 'none' as reference

### Comparative literature

To compare the calculated prevalence and related factors with findings from other studies, six relevant publications were identified for comparison. Detailed data from these retrospective studies are presented and contrasted in Table 4.

**Table 4:**
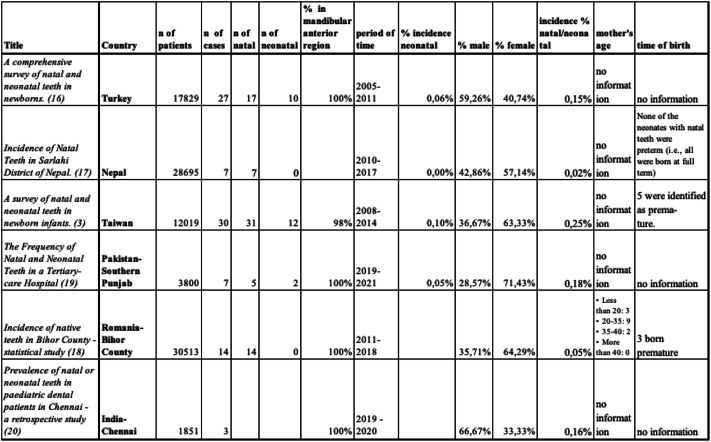
Detailed data on natal and neonatal teeth from retrospective studies chosen for the comparison to the Polish population

## Discussion

To our knowledge, this retrospective study encompasses the longest period of observation for this anomaly published over the last 10 years. In our study, during the 15-year monitoring period, a total of 471 natal/neonatal teeth were observed. The highest incidence was recorded in 2022, with a rate of 0.134%. Some years, such as 2009, showed a marked decrease in incidence: 0.24%. The prevalence consistently increased after 2015, showing an upward trend toward 2022 and statistical analysis has shown a significant rise over time in both males and females. The observed upward trend in the prevalence of these phenomena highlights the need for increased clinical awareness.

The increase in incidence may be attributed to improved detection and reporting, changes in healthcare practices, or actual increases in prevalence due to yet unidentified factors. These may include maternal health conditions such as endocrine or metabolic disorders, supplementation, and drug intake during pregnancy. Additionally, exposure to environmental toxins (e.g., pesticides, endocrine-disrupting chemicals) and air pollution- both of which have been linked to developmental anomalies- may also influence early tooth eruption.

According to the available literature, the largest number of natal/neonatal teeth in retrospective studies was recorded among children born at Kaohsiung Chang Gung Memorial Hospital in Taiwan over a 6-year period with 43 such teeth reported [3]. Covering the same period, overall, 32 natal/neonatal teeth were found in the Neonatal Clinic in Turkey [16]. Both in the Sarlahi district in Nepal and the Oradea County Emergency Clinical Hospital of Obstetrics and Gynecology in Romania [17, 18], data collected over 7 years were evaluated and during this time those facilities registered 7 and 20 natal/neonatal teeth, respectively. Analysis conducted in a hospital located in Southern Punjab, Pakistan revealed 7 cases of such teeth over a two-year period [19], while at the University of Chennai, India − 3 cases between 2019 and 2020 [20].

The large discrepancy between our and other researchers’ results could be related to the fact that our data include registries from the entire voivodeship, the largest structural unit of the state while the previously mentioned retrospective studies referred to single regions or facilities.

Based on data regarding all live births in 2007–2022 in the Greater Poland voivodeship the prevalence of natal and neonatal teeth was determined to be 0.08%. In comparison to studies conducted by other authors, Pogan and coll. in Romania reported a similar incidence ratio of 0.046% [18]. On the other hand, several researchers recorded higher values of natal/neonatal teeth occurrence in their study population. The cases of natal/neonatal teeth accounted for up to 0.25% in the study conducted in Taiwan [3]. Whereas the lowest incidence of this anomaly was observed in Nepal where the result was 0.024% [17]. The analysis of the data revealed that natal teeth were present in 442 (93.8%) of the neonates, while the remaining 29 (6.2%) exhibited neonatal teeth, which erupted within the first 30 days of life. This distribution is consistent with findings from other studies, which highlight a notable predominance of natal teeth over neonatal teeth. Specifically, Pogan et al. and Agrawal et al. did not report any cases of neonatal teeth in their respective cohorts [17, 18]. Additionally, while the presence of neonatal teeth has been documented in several other studies, they occur less frequently than natal teeth, with neonatal teeth comprising 28% to 31% of all recorded cases [3, 16, 19].

According to our data, between 2007 and 2022 the anomaly of natal/neonatal teeth was reported in 256 females. Analysis of the results from other retrospective studies revealed the prevailing allocation of women in the total number of recorded cases. Specifically, women accounted for 57%, 63%, 64% of all cases in the research by Agrawal and coll, Wang and coll, Pogan and coll, respectively [3, 17, 18]. The highest representation of females was noted in a study by Zafar and coll conducted in Pakistan, where women constituted 71% of all cases [19]. In two studies, the incidence of this anomaly was predominantly found among males. In the Indian cohort, males comprised 67% of the sample population, while in the Turkish cohort, the sex distribution revealed natal/neonatal tooth prevalence among males at 59% [16, 20].

Although our results may align with previous studies, none of the studies conducted statistical analysis. In our study, the analysis revealed that while 54% of the anomalies involved women, there were no statistically significant differences when compared to the total number of births. This highlights the need for statistical analyses in future studies conducted in other populations to better understand gender distribution patterns.

The other factor analyzed in our population was the maternal age, with cases distributed across various age groups. While most cases were observed in mothers aged 20–35 years—followed by those under 20 and over 40, statistical analysis revealed that specifically, mothers under 20 and those aged 20–35 had a significantly higher probability of having affected children. In contrast, no significant trend was found for mothers over 35 years of age, indicating that advanced maternal age did not affect the occurrence of these anomalies. Although a similar maternal age pattern was reported in the Romanian study [18], the lack of statistical analysis in other research makes direct comparisons and conclusions about maternal age associations more difficult.

It is important to note that the observation of most cases in mothers aged 20–35 years, in studies lacking statistical analysis may be influenced by the fact that most children are born to mothers aged 20–35 years, reflecting the demographic composition of the childbearing population rather than a specific association between maternal age and the incidence of natal or neonatal teeth. Additionally, as other studies lacked detailed data on maternal age, direct comparisons and interpretations remain challenging.

In our study, the time of birth was categorized as on-time, premature, or post-term. In other research, most natal and neonatal teeth cases were observed in on-time births, with fewer cases reported in premature or post-term deliveries. This trend was also reflected in studies from Nepal and Romania, where most cases were associated with full-term births [17, 18]. Conversely, the Taiwanese study reported a significant proportion of cases (16.7%) among premature infants [3]. However, the predominance of cases in full-term births may simply reflect the fact that most children are naturally born on time. This makes it challenging to establish a specific association between the timing of birth and the occurrence of natal or neonatal teeth. Instead, the higher incidence in full-term births might mirror the overall distribution of birth timings rather than indicating a causal relationship. Further research, supported by thorough statistical analysis is needed to adjust for the natural distribution of birth timings and provide a clearer understanding of the potential influence of prematurity or post-term deliveries on the occurrence of natal and neonatal teeth. Such studies in different populations could help uncover whether specific birth timings contribute to the development of these dental anomalies or if the observed trends are purely demographic.

The reviewed studies provided other clinical and related factors associated with natal and neonatal teeth, emphasizing their multifactorial etiology. Positive family history was reported in multiple cases, with studies such as Wang et al. noting a hereditary component in 16.7% of cases, suggesting a possible genetic predisposition [3]. Tooth mobility was another significant factor, as observed by Zafar et al. and Wang et al., with many cases exhibiting mobility greater than degree I, often necessitating extraction to prevent complications like aspiration or injury [3, 19]. The appearance of natal and neonatal teeth varied, with descriptions of poorly developed, small, conical shapes and hypoplastic enamel or dentin in colors ranging from whitish to yellowish-brown, as noted by Gunasekaran et al. [20]. Riga-Fede disease, caused by trauma from sharp incisal edges, was a frequent complication, with Zafar et al. reporting ventral tongue trauma in all cases, which interfered with feeding and required treatment [19]. Most studies confirmed that these teeth predominantly occurred in the mandibular anterior region and were poorly developed, consistent with findings by Pogan et al. [18].

## Study strengths and limitations

Due to the retrospective design of the study and reliance on anonymized statistical records, detailed clinical information (family history, treatment methods, or other specific characteristics) was unavailable. Additionally, as the analysis was based on registry data, the possibility of underreporting or misclassification cannot be entirely excluded.

At the same time, the study also has notable strengths as it covers a large population size over a long 15-year period, and it is based on standardized government data. These features allowed us to perform reliable statistical analyses and provide accurate incidence of natal and neonatal teeth and how their incidence has changed over time in researched population.

## Conclusions

Collectively, all discussed factors underscore the complexity of natal and neonatal teeth, highlighting the need for careful clinical evaluation of the phenomenon to optimize the diagnosis and appropriate intervention. Moreover, comparative analyses of international research reveal noticeable variations in the reported incidence of natal and neonatal teeth. These differences are likely attributable to differences in methodologies and reporting standards.

This research emphasizes the need for further research employing standardized methodologies and the inclusion of statistical testing in similar population-based studies to enhance the comparability of results across countries and regions.

The findings of this study underscore the value of long-term population monitoring, improving our understanding of how maternal age, timing of delivery, and newborn sex may influence the occurrence of natal and neonatal teeth. This knowledge may contribute to improved diagnosis and care for newborns, as well as providing better support for their caregivers.

## Data Availability

The birth datasets analyzed in this study are publicly available in the Central Statistical Office (GUS) repository at [https://demografia.stat.gov.pl/bazademografia/Tables.aspx] (https:/demografia.stat.gov.pl/bazademografia/Tables.aspx). The data obtained from the National Health Fund (NFZ), are not publicly available. These data may be made available by the authors upon reasonable request and with permission from the National Health Fund.
